# Electricity Consumption Forecasting Scheme via Improved LSSVM with Maximum Correntropy Criterion

**DOI:** 10.3390/e20020112

**Published:** 2018-02-08

**Authors:** Jiandong Duan, Xinyu Qiu, Wentao Ma, Xuan Tian, Di Shang

**Affiliations:** School of Automation and Information Engineering, Xi’an University of Technology, Xi’an 710048, China

**Keywords:** electricity consumption forecasting, least-square support vector machine, maximum correntropy criterion, K-fold cross-validation

## Abstract

In recent years, with the deepening of China’s electricity sales side reform and electricity market opening up gradually, the forecasting of electricity consumption (FoEC) becomes an extremely important technique for the electricity market. At present, how to forecast the electricity accurately and make an evaluation of results scientifically are still key research topics. In this paper, we propose a novel prediction scheme based on the least-square support vector machine (LSSVM) model with a maximum correntropy criterion (MCC) to forecast the electricity consumption (EC). Firstly, the electricity characteristics of various industries are analyzed to determine the factors that mainly affect the changes in electricity, such as the gross domestic product (GDP), temperature, and so on. Secondly, according to the statistics of the status quo of the small sample data, the LSSVM model is employed as the prediction model. In order to optimize the parameters of the LSSVM model, we further use the local similarity function MCC as the evaluation criterion. Thirdly, we employ the *K*-fold cross-validation and grid searching methods to improve the learning ability. In the experiments, we have used the EC data of Shaanxi Province in China to evaluate the proposed prediction scheme, and the results show that the proposed prediction scheme outperforms the method based on the traditional LSSVM model.

## 1. Introduction

With rapid socioeconomic development and the improvement in living standards of residents, the liberalization of the sales side has become an important part of the electricity reform. Electricity selling enterprises have formed a new trading platform and profit model based on the power supply and distribution service. However, the user’s electricity consumption (EC) is easily affected by many factors and shows clear volatility. Therefore, it is necessary for electricity selling companies to make reasonable electricity forecasting systems. For the current bilateral bidding system, electricity selling enterprises buying more or less electricity will face unnecessary economic losses; thus the accuracy requests of electricity demand forecasting are increasingly high.

At present, the methods for electricity consumption prediction (ECPM) can essentially be classified into two types: the traditional prediction method and the intelligent prediction method. In [1], a correction was introduced to correct the coefficients of the Non-homogenous discrete exponential Grey Model (NGM) model, and the raw data was processed with the buffer operator. Finally, a gray prediction model with optimized coefficients was established. However, this model does not consider some of the effects of other factors for electricity. Xue et al. [2] proposed a method of quantifying stochastic change, and its quantized value as a predictive model was used for influencing factors of monthly EC. In addition, the impact of stochastic changes on the monthly sales of electricity was considered reasonable, and thus the model prediction accuracy was effectively improved. An improved support vector regression algorithm was proposed by using the seasonal index adjustment strategy to fine-tune the model prediction error and optimize the key parameters on the basis of the fruit fly algorithm in [3]. A short-term prediction model of building energy consumption based on artificial neural networks (ANNs) and a Bayesian regularization algorithm was developed in [4], and the influence of the parameters such as the delay and the number of hidden neurons was discussed carefully. In addition to the conventional factors such as temperature, the heat island effect as an unconventional factor has also been considered in [5,6,7,8] to improve the accuracy of the prediction. Although these methods above perform well, the following problems should be considered further: (1) Good prediction results mostly depend on a large amount of historical electricity data; however, most users cannot provide enough data; (2) The assumption of the prediction error with a Gaussian distribution is usually used in most of the intelligent algorithms, but it is inconsistent with the diversity of true prediction errors; (3) The prediction results are easily influenced by the type and characteristics of the data.

In order to address these problems, the least-square support vector machine (LSSVM)-based prediction model [9] is introduced to solve the problem of too few data samples effectively. At the same time, the mean square error (MSE) is used as a risk function in the LSSVM model to evaluate the performance obtained by the corresponding parameters [10]. However, the applicability of the MSE to train a mapper (any model mapping an input–output relation, such as neural networks, SVMs, etc.) is optimal only if the probability distribution function of the prediction errors is Gaussian [11,12]. In order to deal with the non-Gaussian and nonlinear problems in engineering applications, a novel criterion, namely, the maximum correntropy criterion (MCC), was introduced in [12,13], and its properties are discussed in [14,15]. Because the prediction errors under the small-sample electricity data have non-Gaussian statistical characteristics, the LSSVM prediction mechanism using the MCC is developed in this paper. Furthermore, the optimization method of grid optimization and the *K*-fold cross-validation method are used to optimize the key parameters of the model, which can ensure the universality of the parameters. Finally, we use the proposed prediction mechanism to predict the EC of large industries in Shaanxi Province, Xi’an City and an educational institution in Xi’an for evaluating its performance.

The rest of the paper is organized as follows: In Section 2, the LSSVM is briefly reviewed. In Section 3, we give the implementation of the LSSVM method based on MCC. In Section 4, we evaluate the accuracy of the proposed model in three cases. Finally, we conclude this paper in Section 5.

## 2. Review of the LSSVM and MCC

### 2.1. Least Square Support Vector Machine

SVM theory is the combination of structural risk minimization and VC dimension theory [16], which are usually utilized for data analysis, pattern recognition, and fitting functions [17,18]. It is superior to other models when dealing with small-sample datasets, nonlinear problems and high-dimensional models. In this section, the LSSVM model is reviewed firstly.

We give a sample set as

(1) $$S=\left\{\left(x_{1},y_{1}\right),\ldots ,\left(x_{i},y_{i}\right)\right\}\subset R^{n}\times R$$

The essence of the regression problem is to search a function *f* corresponding to the sample space, which can map the data from a low-dimensional input space to a high-dimensional output space. The function *f* is a linear function of the variable ω.

The original optimization problem is

(2) $$\min\frac{1}{2}{∥\omega ∥}^{2}$$

Now we define a loss function as

(3) $$I\left(y,f\left(x\right)\right)={\left(y-f\left(x\right)\right)}^{2}=e^{2}$$

where e=y−f(x). The LSSVM can be derived by the following risk function:

(4) $$J_{1}=\frac{1}{2}{∥\omega ∥}^{2}+\frac{1}{2}\gamma \sum_{i=1}^{l}e_{i}^{2}$$

where γ is a penalty.

Then, we can solve the original optimization problem by minimizing Equation (4) as

(5) $$\min J_{1}\left(\omega ,e\right)=\frac{1}{2}{∥\omega ∥}^{2}+\frac{1}{2}\gamma \sum_{i=1}^{l}e_{i}^{2}$$

Now, using Equation (5) and the Lagrange multiplier method, we have

(6) $$L=\frac{1}{2}{∥\omega ∥}^{2}+\frac{1}{2}\gamma \sum_{i=1}^{l}e_{i}^{2}-\sum_{i=1}^{l}\alpha_{i}\left\{\omega^{T}\varphi \left(x_{i}-b+e_{i}-y_{i}\right)\right\}$$

where $\alpha_{i}$ denotes the Lagrange multiplier.

Further, we compute the gradient with respect to each parameter and set it equal to zero, yielding

(7) $$\left\{\begin{array}{c} \frac{\partial L}{\partial \omega }=0\Rightarrow \omega =\sum_{i=1}^{l}\alpha_{i}\varphi \left(x_{i}\right)\\ \frac{\partial L}{\partial b}=0\Rightarrow \sum_{i=1}^{l}\alpha_{i}=0\\ \frac{\partial L}{\partial e_{i}}=0\Rightarrow \alpha_{i}=\gamma e_{i}\\ \frac{\partial L}{\partial \alpha_{i}}=0\Rightarrow b=y_{i}-\omega^{T}\varphi \left(x_{i}\right)-e_{i} \end{array}\right.$$

Now, eliminating ω and *e* in Equation (7), we obtain

(8) $$\left[\begin{array}{cc} 0 & I^{T}\\ I & \Omega +D_{y}^{-1} \end{array}\right]\left[\begin{array}{c} b\\ \alpha \end{array}\right]=\left[\begin{array}{c} 0\\ y \end{array}\right]$$

where the element of the matrix Ω is $\Omega_{ij}=\varphi {\left(x_{i}\right)}^{T}\varphi \left(x_{j}\right)$, which denotes the identify matrix and is the vector of Lagrange multipliers.

After obtaining the variables *b* and α by solving the above Equation (8), we can obtain the regression function as

(9) $$y\left(x\right)=\sum_{i=1}^{l}\alpha_{i}K\left(x_{i},x_{j}\right)+b$$

In the formula, $K(x_{i},x_{j})$ is a kernel function satisfying the Mercer condition [19,20], which can map the feature quantities in the original space into the high-dimensional space. In LSSVM, the commonly used kernel functions include the linear kernel function, the polynomial kernel function, the Gaussian kernel function, the multi-layer perceptron kernel function and so on. Among these, the Gaussian kernel function with its relatively simple structure is famous for its generalization ability and generality [19], and it is defined as

(10) $$K\left(x_{i},y_{i}\right)=\exp\left(-\frac{{∥x_{i}-y_{i}∥}^{2}}{2\sigma^{2}}\right)\;$$

The selection of the kernel function is an important issue for the SVM. For the process of forecasting electricity, the data is usually stored in a low-dimensional vector space, which may lead to a difficulty in extracting hidden information from the data. After continuous reasoning of mathematics, a feasible method for the classification of low-dimensional vector sets is to map the low-dimensional-space vector number set to a high-dimensional space, although the computation complexity will be increased. In order to simplify the calculation steps, the kernel function was introduced for SVMs to solve the mapping problem. SVMs map the sample space to a high-dimensional space by nonlinear mapping, which transforms the nonlinear separable problem in the original sample into a linear separable problem in high-dimensional space, which is called dimension increase and linearization.

### 2.2. Maximum Correntropy Criterion

Considering the non-Gaussian distributions feature of the EC, a suitable risk function minimizing the information content of the error distribution instead of minimizing the MSE should be better employed to guide the parameter optimization process, then improving the electricity prediction accuracy efficiently. The MCC as a measure of the information content in information theoretic learning was developed by principe with his team to deal with error distributions with non-Gaussian characteristics, and it has been widely used in pattern classification, feature selection, dimension reduction and adaptive filtering [21,22,23,24,25]. The MCC indicates the similarity between the predicted output and the real sample in the correntropy sense; it shows good robustness for nonlinear and non-Gaussian data processing, such as determining whether electricity is suitable for the prediction of time series non-stationary and time-varying predictions [26]. The correntropy between two arbitrary random variables *X* and *Y* is defined by

(11) $$\begin{array}{cc} V_{\sigma }\left(X,Y\right) & =E\left[G_{\sigma }\left(X-Y\right)\right]\\ & =\int_{}G(e)f_{e}\left(e\right)de \end{array}$$

where e=x−y is the error, $G_{\sigma }\left(\cdot \right)$ denotes the kernel function with the kernel width σ, and E(·) represents the mathematical expectation. In practice, the joint distribution of *X* and *Y* is usually unknown, and only a finite number of samples of these are available as ${\left\{\left(x_{i},y_{i}\right)\right\}}_{i=1}^{m}$; this leads to the following sample estimator of correntropy using the Parzen window estimate [12] as follows:

(12) $$V_{\sigma }\left(X,Y\right)=\frac{1}{N}\sum_{i=1}^{N}G_{\sigma }\left(x_{i}-y_{i}\right)$$

where $G_{\sigma }\left(x_{i}-y_{i}\right)=\exp\left(-\frac{{∥x_{i}-y_{i}∥}^{2}}{2\sigma^{2}}\right)$ is a Gaussian kernel function, and we know that the value of Equation (12) is always positive and obtains its maximum when X=Y.

As mentioned above, the correntropy uses the high-order moment of the signal, which can be represented by the Taylor series expansion of the Gaussian kernel function:

(13) $$V_{\sigma }\left(X,Y\right)=\frac{1}{\sqrt{2\pi \sigma }}\sum_{n=0}^{\infty }\frac{{\left(-1\right)}^{n}}{2^{n}\sigma^{2n}n!}E\left[{\left(X-Y\right)}^{2n}\right]\;$$

Remark: As one can see, when using the Gaussian kernel function, correntropy contains all even-order sums of random variables *X* and *Y* [12,14]. By using the above index, the MCC contains the high-order moment between the real value and the predicted value, and it is mostly guided by the local similarity of the data. For the EC forecasting problem, if the overall similarity is only considered while the local similarity is ignored, it is possible to meet the requirements of the local and global error caused by a large and unacceptable error. Therefore, the introduction of the MCC, producing local similarity between real data and forecast data as an evaluation index, is more reasonable than the MSE, which gives global similarity.

## 3. FoEC via LSSVM with MCC

On the basis of the theory’s foundation reviewed in Section 2, we design the FoEC method via the LSSVM model with the MCC in this section. For the proposed FoEC, the details of the steps are described as follows:

Step 1: Data preprocessing: Includes the processing of error data and data normalization.
Step 2: Dataset constructing: The normalized data samples are divided into training and testing samples, which are used to train the LSSVM model and evaluate the performance of the trained model, respectively.
Step 3: Parameter optimization: The parameters of the LSSVM model with the MCC are optimized by the grid search method.
Step 4: Prediction: After training the LSSVM, the prediction accuracy and the generalization performance are demonstrated by the testing data.
Step 5: Prediction result analysis: Applies certain evaluation criteria for performing evaluation tasks and analyzing the various elements that affect the result of such a forecast.

We now give the detailed analysis of the prediction procedures in the following subsections.

### 3.1. Data Preprocessing

The collection and the preprocessing of historical data are very important for the prediction scheme. However, some missing or unreasonable and even wrong data are usually contained in the original dataset, which is often obtained by a statistical machine and artificial classification. Accordingly, we should perform analysis and preprocessing of the original data to eliminate the influence of the abnormal data in order to improve the accuracy of the prediction scheme.

(1) Error data and missing data processing.

On the basis of the analysis of the distribution of the EC data, we use the data at the same time point in the last year to replace the error data or the missing data.

For the error data and the missing data in a dataset, these can be replaced by that of the same period of the last year’s electricity and the last or the next month’s electricity of the same year.

(2) Normalization processing.

In our proposed prediction scheme, four variables are considered as the factors affecting the electric power consumption with different dimension. Therefore, the data should be normalized by a suitable method first. In this work, we use the following formula to normalize the data:

(14) $${y_{i}}^{\prime }=\frac{y_{i}-y_{\min}}{y_{\max}-y_{\min}}$$

where $y_{i}$ denotes the electricity data before normalization, and $y_{\max}$ and $y_{\min}$ are the maximum and minimum data in the dataset, respectively; ${y_{i}}^{\prime }$ stands for normalized data.

We can easily obtain the anti-normalization data by the following formula:

(15) $$y_{i}=\left(y_{\max}-y_{\min}\right)*{y_{i}}^{\prime }+y_{\min}$$

### 3.2. Selection of Influence Factors

**Figure 1:**
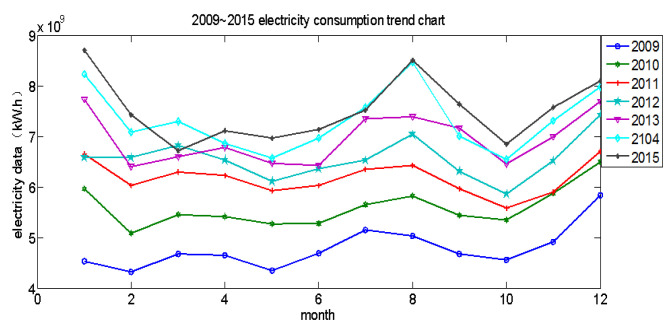
2009–2015 electricity consumption trend chart.

In this work, we mainly focus on predicting the EC of Shaanxi Province, China by using the proposed model. We have collected the EC data each month from 2009 to 2015 in Shaanxi Province. The trend of the EC is given in Figure 1. One can observe that although the EC fluctuated among the different years, the trend of the EC for every year was essentially identical. This primary result indicates that the EC has a certain regularity, and it is predictable.

The main factors affecting EC are the economy, climate, electricity structure, electricity and so on. After analyzing the existing data, from the point of view that it can best reflect the change in EC, this paper chooses historical EC data, gross domestic product (GDP), and the regional temperature for a total of three features supplemented by holidays to correct.

(1) The quantity of electricity data.

According to the historical electricity data analysis above, we can roughly observe the development trend of the electricity demand. Consequently, it provides a profitable tool for extracting rules from that experience and knowledge to estimate the overall distribution trend in the future.

Electricity data always contains hidden information; from these historical data can be summed up the law of changes in demand for electricity; researchers on the basis of these laws can estimate the future trend of the overall distribution of the EC. In order to describe the trend of EC more clearly, this section uses the original quantity of electricity data, which is not normalized.

The historical EC is shown in Figure 1. Horizontally, there is a significant fluctuation in the amount of electricity for the 12 months of the year. First, we calculate the standard deviation of the data by the following Equation (16), and the degree of fluctuation as the proportion of the standard deviation and average value are defined. The maximum fluctuation using Equation (16) of the above data is 23.4%. Longitudinally, the amount of electricity consumed increases to different degrees every year, with an average increase of 5.21%.

(16) $$RMSE=\sqrt{\frac{1}{N}\sum_{i=1}^{N}{\left(x_{i}-\bar{x}\right)}^{2}}$$

where *N* denotes the number of the month, $x_{i}$ is the monthly electricity, and $\bar{x}$ is the average annual EC.

(2) Regional temperature.

**Figure 2:**
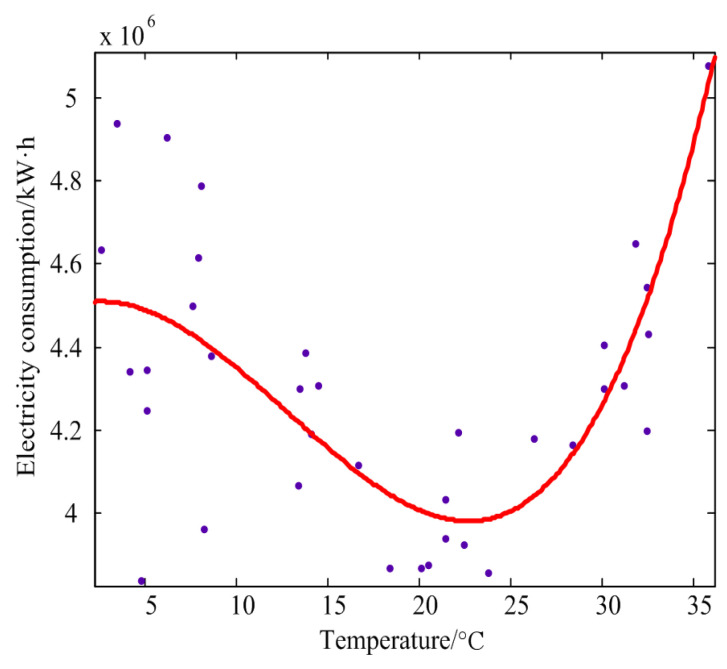
Scatter diagram of monthly average temperature and monthly electricity consumption.

For the current residential or industrial EC, there is a relationship between the temperature and the EC [27], as shown in Figure 2. In the season with a high temperature, the user’s power consumption will be greatly increased as a result of the input of refrigeration facilities such as air conditioners, and in the season with low temperature, the warmth will still consume a large amount of electric energy. Therefore, this paper also sets the temperature as an important factor affecting the EC.

(3) GDP.

Economic factors, such as the development of industry and commerce, have an influence on the power system, because the rapid development of industry and commerce necessarily leads to an increase in electricity demand. In this paper, we use the GDP, which is the most representative in the field of economy, to represent economic factors.

(4) Number of holidays and types of holidays.

Holidays have significant impacts on the electricity demand. In general, holiday production will be significantly reduced and electricity demand will decrease accordingly. However, the demand of electricity may increase in the Spring Festival and on the National Day because of residential electricity use substantially increasing. Accordingly, we should conduct an analysis of the characteristics of the local electricity demand to confirm the change in its trend (i.e., increasing or decreasing). Finally, after predicting the user’s EC using the proposed scheme, the prediction result may also be corrected according to the holidays.

### 3.3. Parameter Optimization

When the LSSVM model is used find to the quantity of electricity, the kernel parameters $\sigma^{2}$ and the regularization parameter γ have a very important effect for the prediction accuracy; thus they need to be optimized firstly. The grid optimization method and *K*-fold cross-validation are combined to optimize the parameters in this paper. The reasons and principle introduction are as follows:

Using this combination algorithm is simple and direct for searching the parameters of LSSVM, and the searching speed compared with the test-set parameters may be faster.

The main mechanism of the combination algorithm combination?algorithm is to divide the parameters in a predetermined range into a certain number of grids and then make the model traverse all the parameters in the grid to determine the parameters of the model performance.

Just as for the traditional LSSVM model, two parameters in the proposed ECPM need to be optimized. During the optimization process, we used the training samples to train the LSSVM model, while the MCC instead of the MSE was employed to evaluate the optimal performance obtained by the certain parameters in the set of parameters $\sigma^{2}$ and γ.

In order to perform the parameter optimization, the MCC is introduced as a risk function. When the training sample does not satisfy the Gaussian distribution, it can also find the appropriate parameters, which is very helpful to improve the accuracy of the prediction of the quantity of electricity. The MCC can be expressed by Equation (17):

(17) $$\mathrm{MCC}\Leftrightarrow \frac{1}{m}\sum_{i=1}^{m}\exp(-\frac{{∥a_{i}-b_{i}∥}^{2}}{2\sigma_{0}^{2}})$$

where $\sigma_{0}$ is the value of the kernel width optimized by the mesh optimization method.

**Figure 3:**
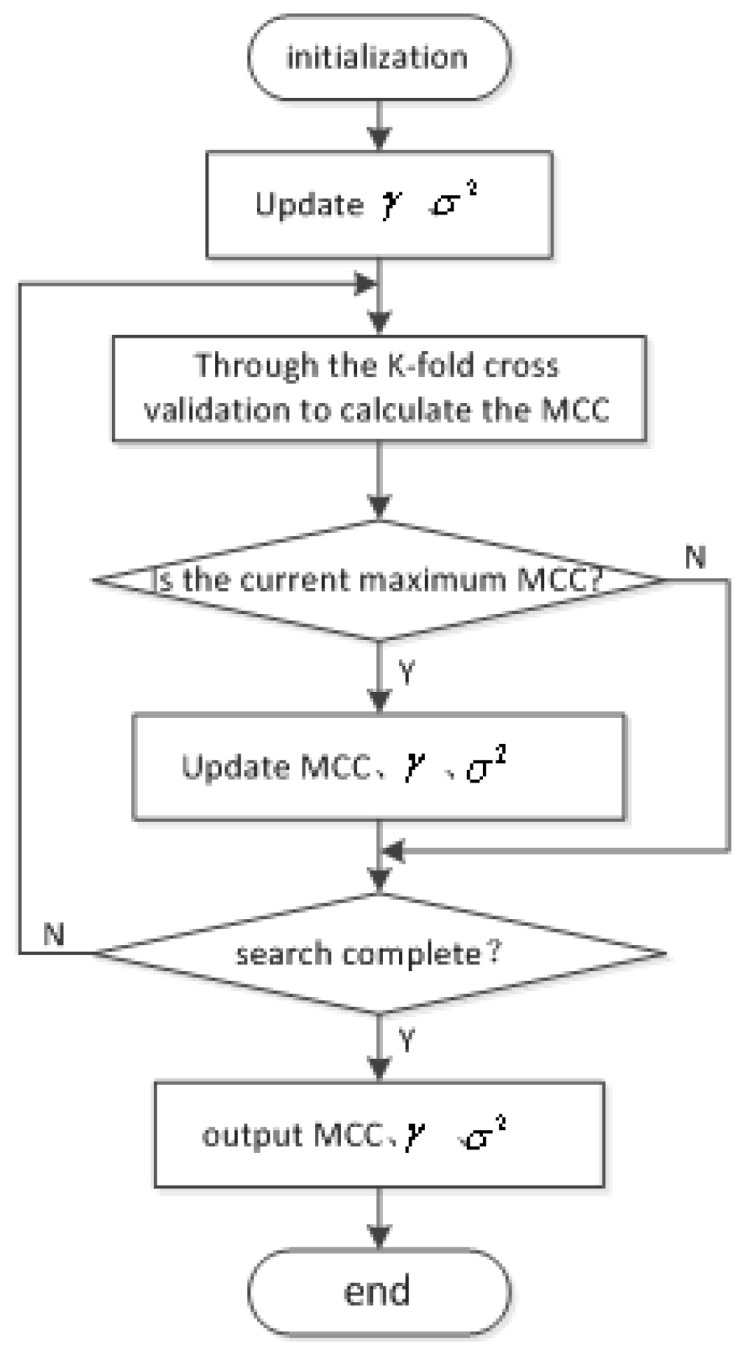
Parameter optimization flow chart.

By Equation (17), we conclude that the larger the value of the MCC, the greater the similarity of the data between the predictive value and the real value. To this end, we select the parameters that make the MCC the maximum as the optimal parameter of the prediction model. The flow chart of the parameter optimization is shown in Figure 3.

### 3.4. Performance Evaluation Function (PEF)

The whole quantity forecast should also include the evaluation of the forecast results. From the rigor of the study, using a single error indicator to evaluate the results of the prediction is not reliable. As a result, we choose the mean relative error (MRE), correlation coefficient (*R*), and the maximum prediction error ($\delta_{\max}$) as the error statistical index to evaluate the performance of the proposed prediction scheme, and the definitions of these are as follows:

(18) $$MRE=\frac{1}{n}\sum_{i=1}^{n}\frac{\left|y_{i}-y_{pi}\right|}{y_{i}}$$

(19) $$R=\sqrt{1-\frac{\sum {\left(y_{pi}-y_{i}\right)}^{2}}{\sum {\left(y_{i}-\bar{y}\right)}^{2}}}$$

(20) $$\delta_{\max}=\max\left(\left|y_{pi}-y_{i}\right|\right)$$

where *N* stands for 12 months, $y_{pi}$ is the forecasting electricity for every month, $y_{i}$ is the true electricity of every month, and $\bar{y}$ is the average annual EC.

## 4. Prediction Results and Analysis

In this section, we have performed an experiment using Matlab2013 and the LSSVM toolkit to evaluate the prediction performance of the proposed model. Experimental data is from the national grid company of Shaanxi Province from 2009 to 2015 with a period of 1 month. The prediction accuracy of the electric quantity is influenced by many factors, including historical data, GDP growth, meteorological factors and so on.

In the forecasting of the electricity, all kinds of factors that affect the demand of electricity are analyzed. In order to find out how the factors affect the electricity demand forecasting, we obtained forecasting results by only considering electricity data first, and then obtained further prediction results that considered other factors. Two prediction datasets were used to perform the contrast analysis.

For only using the historical electricity data to make predictions, the data samples needed to be divided into prediction samples and training samples. The length of the training samples had to also be divided naturally. In the setting of the experiment, we used the data of 2009–2014 as the training sample. The training data samples were divided into four groups; in other words, we could train four times by using 6 years worth of data, as shown in Equation (21), by using the first 2 years of electricity data to predict the data of the third year, through increasing the number of training times to make up for the reduction in the data dimension.

(21) $$\left\{\begin{array}{c} y_{2011}=f_{1}\left(x_{2009},x_{2010}\right)\\ y_{2012}=f_{2}\left(x_{2010},x_{2011}\right)\\ y_{2013}=f_{3}\left(x_{2011},x_{2012}\right)\\ y_{2014}=f_{4}\left(x_{2012},x_{2013}\right) \end{array}\right.\Rightarrow y_{2015}=f\left(x_{2013},x_{2014}\right)\;$$

### 4.1. Prediction Results of Large Industry in Shaanxi Province

In this section, we forecast the electricity of a large industry in Shaanxi Province that is closely related to people’s lives, to research the impact of different industries on the EC using MCC–LSSVM and MSE–LSSVM. The data comes from the electricity data of the large industrial power consumption in Shaanxi Province.

**Figure 4:**
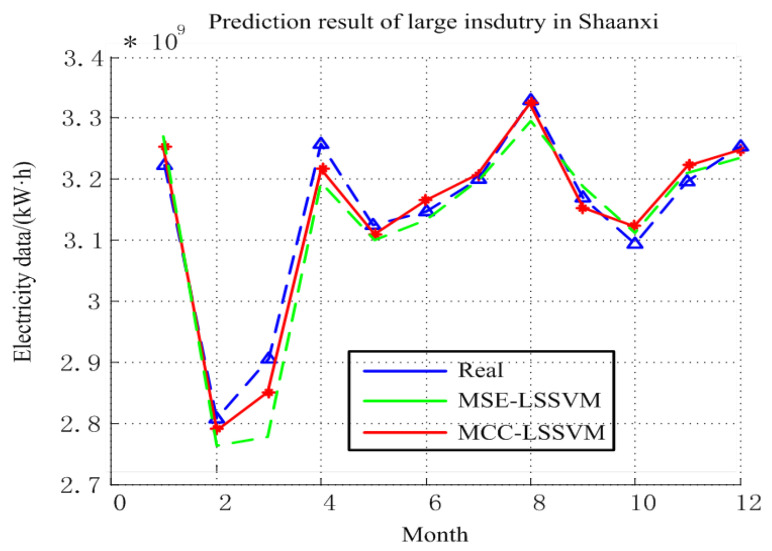
Prediction results for large industry in Shaanxi Province.

The prediction results for the large industry in Shaanxi Province are shown in Figure 4, and Table 1 and Table 2. From Table 1 and Table 2, we can clearly see that the prediction error using MCC–LSSVM was 0.9% and using MSE–LSSVM was 3.12% throughout the year. We can conclude that the prediction effect of the proposed model for large industrial EC is desirable, and the reasons for this result are as follows: First, Shaanxi Province belongs to the inland city, its industrial development is relatively stable, and EC data is neat. Second, the development of the large industry and the growth of the GDP are closely related; the correlation between the two is very high, and thus the GDP factors as a result of the impact of large industrial power factors can achieve better results.

**Figure 5:**
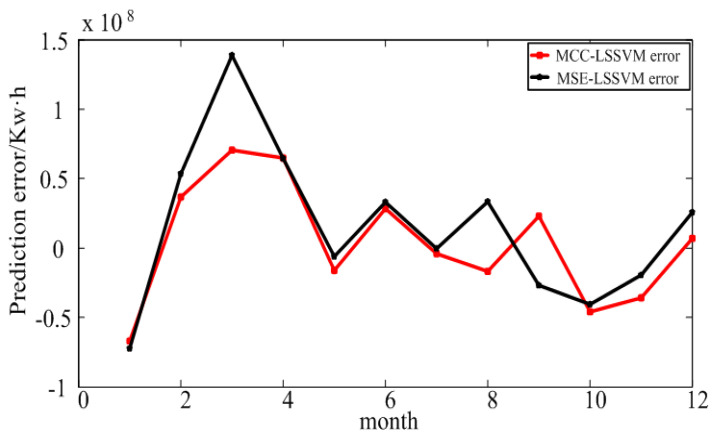
Prediction error for large industry in Shaanxi Province.

From Figure 5, one can see that the MCC–LSSVM model and MSE–LSSVM model effectively predict the regional EC. However, when using the MSE model, an error outlier appeared in March; the MCC–LSSVM model effectively suppressed the occurrence of this error outlier.

### 4.2. Prediction Result for Xi’an

In order to verify the reliability of the proposed MCC–LSSVM model, we forecast the EC in Xi’an City, Shaanxi Province.

**Figure 6:**
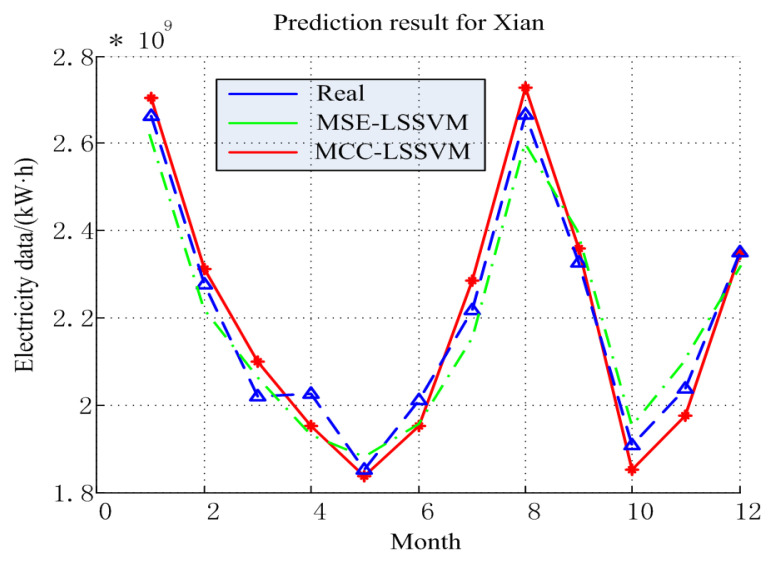
Prediction result for Xi’an.

**Figure 7:**
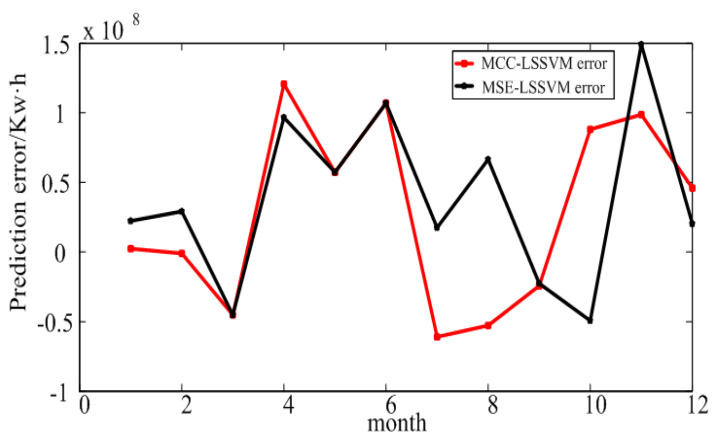
Prediction error for Xi’an.

The MCC–LSSVM and MSE–LSSVM model prediction results and error of the power consumption in 2015 are shown in Figure 6 and Figure 7. The detailed results are given in Table 3 and Table 4. From Figure 6, one can see that the prediction result of Xi’an City was worse than for the large industry in Shaanxi Province. By Equation (18), using the MCC–LSSVM model, we obtained the annual average relative error for Xi’an of 2.77%, and the MSE–LSSVM model produced 3.23%. We explain the above results by the following two points: First, the Shaanxi Province electricity structure is more optimal, such that the power consumption data of Shaanxi Province is by contrast neater. Second, we chose the GDP as an influence factor in this experiment. As is known to all, the GDP is mainly contributed to by the manufacturing by machines; however, Xi’an is a service industry-based city. Therefore, the correlation between the GDP factor and Xi’an’s electric power development is not very strong.

### 4.3. Prediction Results of Electricity Consumption in an Educational Institution in Xi’an

**Figure 8:**
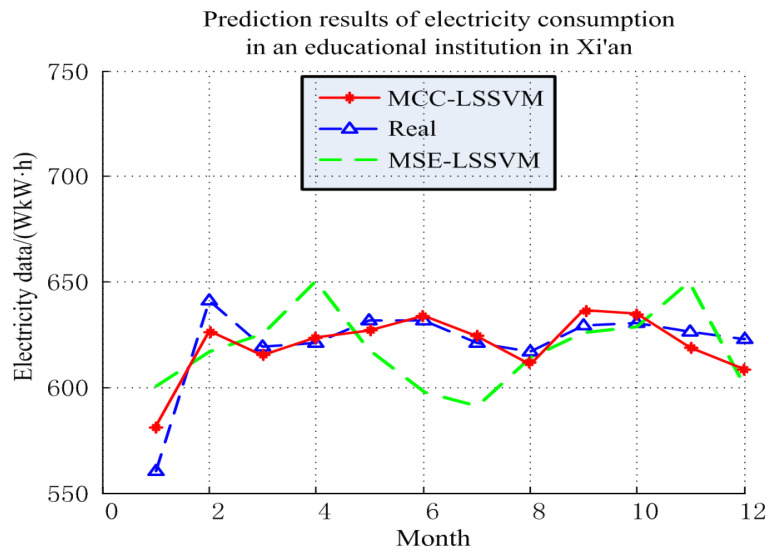
Prediction results of electricity consumption in an educational institution in Xi’an.

**Figure 9:**
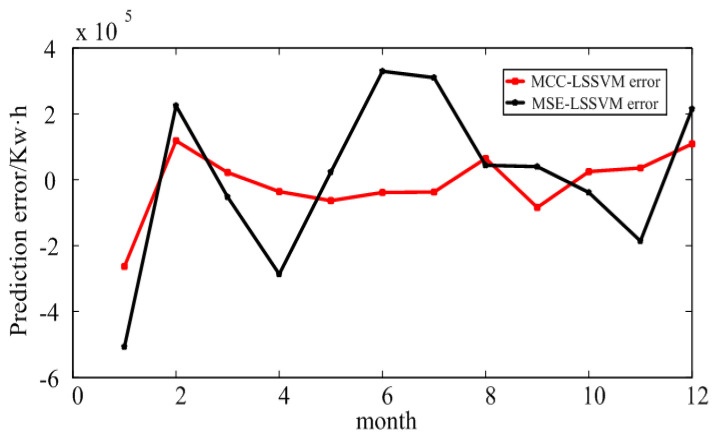
Prediction error of electricity consumption in an educational institution in Xi’an.

The electricity prediction results and error in an educational institution are shown in Figure 8 and Figure 9, and Table 5 and Table 6. According to these results, one can conclude that the MCC–LSSVM model describes the change trend of the history of the electricity more accurately than the MSE–LSSVM model, and it makes an accurate prediction of the future EC. Using the above two models to predict the EC in an educational institution, we found that the prediction error of the MCC–LSSVM model was 3.98%, and the prediction error of the MSE–LSSVM model was 6.41%. At the same time, the MCC–LSSVM model effectively avoided the situation in which local errors were too large in June and July.

**Figure 10:**
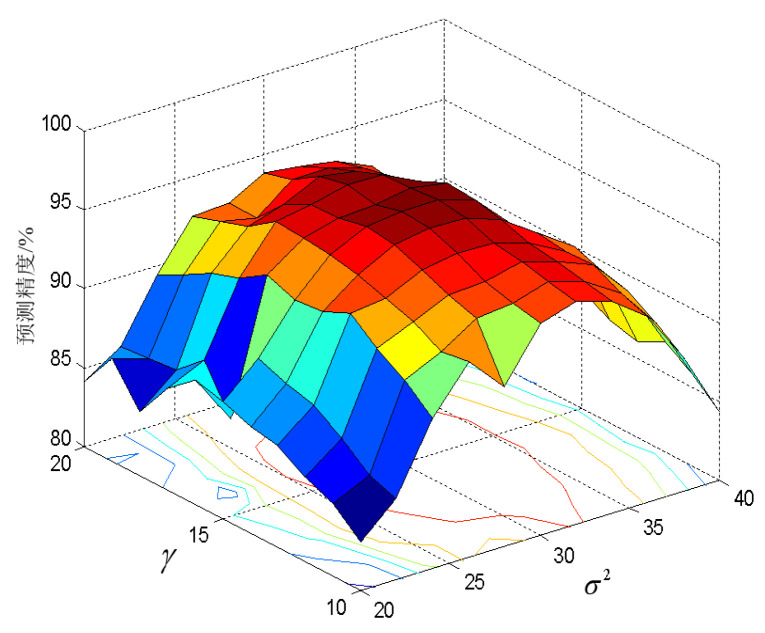
Three-dimensional map of prediction accuracy, varying with parameters γ and $\sigma^{2}$.

At the same time, the two key parameters of the MCC–LSSVM model are γ=15 and $\sigma^{2}=31$. In the previous article, the selection of γ and $\sigma^{2}$ had a great impact on the prediction accuracy. As can be seen from Figure 10, in the [10 15] interval of γ and for $\sigma^{2}$ in the [20 31] interval, the increase in γ and $\sigma^{2}$ increases the prediction accuracy accordingly. When γ and $\sigma^{2}$ are out of the above ranges, increasing the value of these two parameters will cause varying degrees of loss in the accuracy of the prediction.

## 5. Conclusions

Aiming to address the current situation of the non-Gaussian characteristics in the prediction of EC, the MCC as the risk function of the LSSVM, which can effectively avoid the local error, is introduced in this paper. Furthermore, the grid optimization method and *K*-fold cross-validation method with shorter computational times and relatively faster search speeds are utilized to search two key parameters of the proposed model. The prediction mechanism has been evaluated for a large industry in Shaanxi Province, Xi’an City and an educational institution in Xi’an. The results show that the proposed prediction mechanism has a certain guiding value for the electricity side in formulating the electricity purchase plan and the user pricing.

## Figures and Tables

**Table 1:** Prediction results for large industry in Shaanxi Province.

Month	Real/kWh	MCC–LSSVM/kWh	MSE–LSSVM/kWh
1	3222141989	3289063235	3294523968
2	2807359588	2770660550	2753838956
3	2905625349	2835276855	2766953282
4	3258028408	3193065850	3193625474
5	3123940121	3140143741	3130143486
6	3146763568	3118571548	3113648647
7	3200783187	3204753934	3201236988
8	3330358001	3347137347	3297138647
9	3169671810	3146624356	3196624769
10	3094490648	3140563342	3134963398
11	3197240745	3233100710	3216824659
12	3253492846	3246565684	3227682398

**Table 2:** Evaluation index.

Evaluation Index	MRE (%)	$\delta_{\max}$/kWh	*R*
MCC–LSSVM	0.9	73256684	0.9235
MSE–LSSVM	3.12	140348494	0.8952

**Table 3:** Prediction result for Xi’an.

Month	Real/kWh	MCC–LSSVM/kWh	MSE–LSSVM/kWh
1	2664166276	2661798254	2641798254
2	2275553927	2276537980	2246537980
3	2021181824	2066396013	2066396013
4	2025719576	1904917602	1928943561
5	1850231091	1792863625	1792863625
6	2011974726	1904917602	1872354896
7	2215976398	2276963182	2192662853
8	2664937423	2717665734	2596348624
9	2326022304	2350413608	2348629858
10	1906840712	1818905227	1956189345
11	2038734324	1940165576	1889654236
12	2350000000	2303946621	2329946654

**Table 4:** Evaluation index.

Evaluation Index	MRE (%)	$\delta_{\max}$/kWh	*R*
MCC–LSSVM	2.77	120801974	0.9534
MSE–LSSVM	3.23	145653478	0.9316

**Table 5:** Prediction results of electricity consumption in an educational institution in Xi’an.

Month	Real/kWh	MCC–LSSVM/kWh	MSE–LSSVM/kWh
1	5526468	5789523	6034028
2	6435286	6317452	6211205
3	6215832	6194268	6268253
4	6231532	6267145	6518210
5	6231102	6294423	6207253
6	6315468	6354652	5986242
7	6221536	6258553	5912131
8	6189358	6124125	6145128
9	6294825	6378632	6255368
10	6314653	6290058	6353895
11	6277436	6219389	6503896
12	6231862	6123568	6017658

**Table 6:** Evaluation index.

Evaluation Index	MRE (%)	$\delta_{\max}$/kWh	*R*
MCC–LSSVM	3.98	2635648	0.9619
MSE–LSSVM	6.41	3296821	0.9106
